# Esophageal Stent in Acute Refractory Variceal Bleeding: A Systematic Review and a Meta-Analysis

**DOI:** 10.3390/jcm13020357

**Published:** 2024-01-09

**Authors:** Busara Songtanin, Chanaka Kahathuduwa, Kenneth Nugent

**Affiliations:** Department of Internal Medicine, Texas Tech University Health Sciences Center, Lubbock, TX 79430, USA; chanaka.kahathuduwa@ttuhsc.edu (C.K.); kenneth.nugent@ttuhsc.edu (K.N.)

**Keywords:** variceal bleeding, self-expandable metal stents, refractory variceal bleeding, upper gastrointestinal bleeding, cirrhosis, endoscopy

## Abstract

**Background:** Acute esophageal variceal bleeding accounts for up to 70% of upper-gastrointestinal bleeding in cirrhotic patients. About 10–20% of patients with acute variceal bleeding have refractory bleeding that is not controlled by medical or endoscopic therapy, and this condition can be life-threatening. Balloon tamponade is a long-standing therapy which is only effective temporarily and has several complications, while transjugular intrahepatic portosystemic shunt (TIPS) and liver transplantation may not be readily available at some centers. The use of self-expandable metal stents (SEMSs) in refractory esophageal variceal bleeding has been studied for effectiveness and adverse events and has been recommended for use as a bridge to a more definitive treatment. **Aim:** To investigate the effectiveness and safety of SEMSs in managing refractory variceal bleeding. **Methods:** A systematic search of the MEDLINE, EMBASE, and Cochrane library databases was performed from inception to October 2022 using the following terms: “esophageal stent”, “self-expandable metal stents”, “endoscopic hemostasis”, “refractory esophageal varices”, and “esophageal variceal bleeding”. Studies were included in the meta-analysis if they met the following criteria: (1) patients’ age older than 18 and (2) a study (or case series) that has at least 10 patients in the study. Exclusion criteria included (1) non-English publications, (2) in case of overlapping cohorts, data from the most recent and/or most appropriate comprehensive report were collected. DerSimonian–Laird random-effects meta-analysis was performed using the meta package in R statistical software(version 4.2.2). **Results:** Twelve studies involving 225 patients with 228 stents were included in the analyses. The mean age and/or median age ranged from 49.4 to 69 years, with a male-to-female ratio of 4.4 to 1. The median follow-up period was 42 days. The mean SEMS dwell time was 9.4 days. The most common cause of acute refractory variceal bleeding in chronic liver disease patients included alcohol use followed by viral hepatitis. The pooled rate of immediate bleeding control was 91% (95% CI 82–95%, *I*^2^ = 0). The pooled rate of rebleeding was 17% (95% CI 8–32%, *I*^2^ = 69). The pooled rate of stent ulceration was 7% (95% CI 3–13%, *I*^2^ = 0), and the pooled rate of stent migration was 18% (95% CI 9–32%, *I*^2^ = 38). The pooled rate of all-cause mortality was 38% (95% CI 30–47%, *I*^2^ = 34). **Conclusions:** SEMSs should be primarily considered as salvage therapy when endoscopic band ligation and sclerotherapy fail and can be used as a bridge to emergent TIPS or definitive therapy, such as liver transplantation.

## 1. Introduction

Acute esophageal variceal bleeding accounts for up to 70% of upper-gastrointestinal bleeding in cirrhotic patients [1]. The standard treatment (after resuscitation) includes pharmacological therapy with vasoactive drugs (octreotide, somatostatin, and terlipressin); endoscopic variceal band ligation or sclerotherapy should be performed within 12 h along with the initiation of prophylactic antibiotics [2,3,4].

Approximately 10–20% of patients with acute variceal bleeding have refractory bleeding uncontrolled by medical or endoscopic therapy, and this condition can be life-threatening. Balloon tamponade is an easy and readily available technique that is temporarily effective and can provide control in up to 80% of these cases; however, it should not be used for more than 24 h and often leads to complications, such as esophageal tears and perforation [3,5]. Transjugular intrahepatic portosystemic shunts (TIPSs) and liver transplantation are definitive therapy in the management of refractory variceal bleeding. However, TIPSs require technical expertise, and this procedure can increase the frequency of hepatic encephalopathy [6].

Self-expandable metal stents (SEMSs) are devices that can be used in both benign and malignant disorders in the gastrointestinal tract [7]. The use of fully covered SEMSs in refractory variceal bleeding has been studied for effectiveness and adverse events. The current Baveno consensus recommends using balloon tamponade or SEMSs as bridge therapy in centers that do not have definitive treatment readily available; SEMSs are as effective as balloon tamponade and are safer, but these two techniques only provide temporary management before definitive treatment with TIPS or transplantation [4]. A multicenter randomized controlled study compared outcomes in patients with acute refractory variceal bleeding treated with either SEMSs (n = 13) or balloon tamponade (n = 15) in temporary control of acute refractory esophageal variceal bleeding and reported that SEMSs have higher efficacy in the control of bleeding (85% vs. 47%; *p* = 0.037) and have fewer complications (15% vs. 47%; *p* = 0.077) than balloon tamponade in the control of bleeding [8]. 

This meta-analysis updates a prior meta-analysis published in 2020 [9] and includes three additional studies published in 2021 and 2022 [10,11,12] to evaluate the effectiveness and safety profiles of SEMSs in managing acute refractory variceal bleeding, including immediate bleeding control rate, rates of rebleeding, stent ulceration, stent migration, and overall mortality rate as a bridge to more definitive therapy.

## 2. Materials and Methods

### 2.1. Literature Search

PRISMA statement guidelines were followed for conducting and reporting meta-analysis data [13]. A comprehensive literature search of MEDLINE, EMBASE, and Cochrane Library was conducted from inception to October 2022. Two reviewers screened titles and abstracts of all the identified articles that evaluated the performance of SEMSs in refractory variceal bleeding. The MeSH terms for MEDLINE and Cochrane were ((esophageal stent) OR (self-expandable metal stents)) AND ((refractory esophageal varices) OR (esophageal variceal bleeding)). EMBASE search terms were (esophageal and stent OR (‘self-expandable’ AND metal AND stents) OR (endoscopic AND hemostasis)) AND (refractory AND esophageal AND varices OR (esophageal AND variceal AND bleeding)) present in titles, abstracts, or full-text articles.

### 2.2. Inclusion and Exclusion Criteria

Only studies investigating the use of SEMS placement for acute refractory variceal bleeding were included. Only human subject studies were included in the analysis with adult patients (>18 years), and only English studies were included. Case series and reported studies that have fewer than ten patients included in the study were excluded. In studies with overlapping cohorts, data from the most recent and/or most appropriate comprehensive report were included. Systematic reviews, reviews, editorials, conference abstracts, and articles with incomplete data were excluded.

### 2.3. Data Extraction

Data were extracted from the selected manuscripts into pre-defined data fields. The number of stents used in each study, the number of cases with successful control of bleeding and cases with rebleeding, ulceration, and stent migration were extracted to compute the proportions. The total number of participants and the number of deaths were recorded to compute the mortality rates. In addition, the year of publication, mean age of participants, proportion of males, and duration of follow-up were extracted to include in meta-regression analyses (Supplementary Table S1) to determine any moderator effects of these variables on the examined proportions.

### 2.4. Outcome Measures

The primary outcome measurement in this study was the efficacy of the SEMS in controlling acute refractory variceal bleeding. Secondary outcomes included the complication rate (rebleeding, stent migration, and stent ulceration) and the overall mortality rate.

### 2.5. Statistical Analysis

Separate DerSimonian–Laird random-effects meta-analyses were performed using the ‘meta’ package (version 6.5-0) in R statistical software (version 4.3.0) to examine the pooled proportions of achieving bleeding control and the complications of rebleeding, ulceration, and stent migration. An additional meta-analysis was performed to examine the pooled mortality rate [14]. The consistency of the findings of the meta-analyses was confirmed by leave-one-out sensitivity analyses. The likelihood of publication bias was analyzed using funnel plots and Peter’s weighted linear regression [15]. The effect sizes of missing (i.e., unpublished/unreported) studies were imputed using the trim-and-fill method. The heterogeneity of effect sizes was quantified by calculating the Higgins’ I^2^ statistic [16,17]. To explain the heterogeneity of the studies, exploratory univariate random-effects meta-regression analyses were performed to examine the moderator effects of each of the covariates described above [18]. The Newcastle–Ottawa Scale was used for retrospective studies to assess the quality (Supplementary Table S2) [19].

## 3. Results

### 3.1. Search Results and Characteristics of the Included Studies

**Figure 1:**
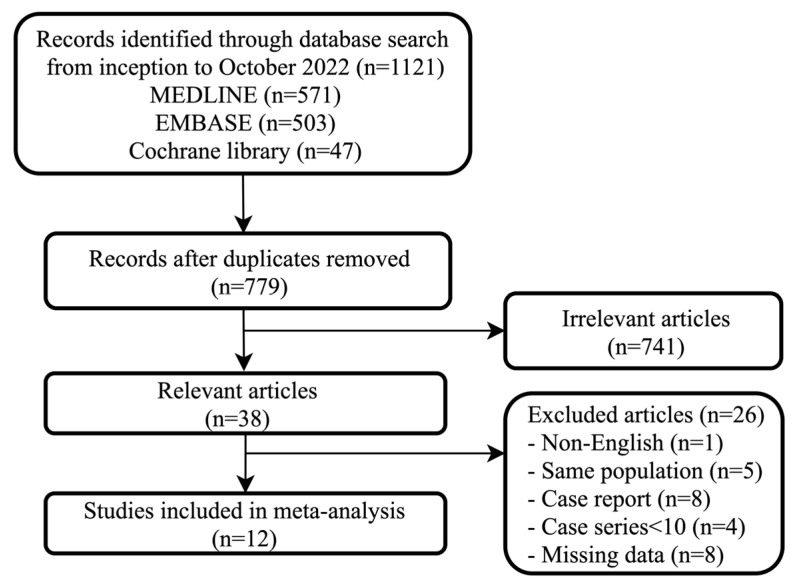
Search strategy diagram according to PRISMA flow chart.

Figure 1 provides a graphical representation of the study screening and selection process according to the PRISMA flow chart. A total of 1121 articles were found using the above search criteria. After the removal of duplicates, a total of 779 articles were found. Of these, 38 studies were found to be relevant. Results of twelve studies published between 2008 and 2021, representing data from 225 patients (male 183 and female 42) presenting with variceal bleeding requiring a total of 228 esophageal stents, were included in the analyses. The mean age of the participants included in the studies ranged from 46.9 to 69 years, and the proportion of males ranged from 50% to 100%. The duration of follow-up in the studies ranged from 30 days to 180 days with a median follow-up of 42 days across studies. Patient characteristics of all included studies are summarized in Table 1.

### 3.2. Successful Controlling of Bleeding

**Figure 2:**
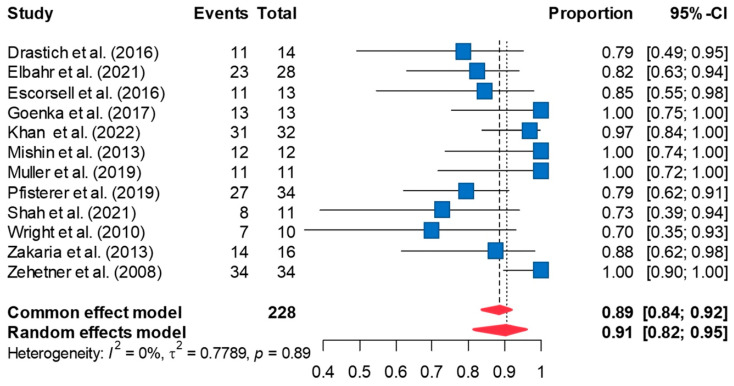
The forest plot of the efficacy of SEMSs in immediate bleeding control demonstrates the rate of successful control bleeding is 90.6% [95%CI 81.8, 95.4] [8,10,11,12,20,21,22,23,24,25,26,27].

The definition of successful control of bleeding is the absence of bleeding within 24 h of SEMS placement (or immediate bleeding control rate). Random-effects meta-analysis including 12 studies revealed successful control of bleeding in 90.6% of the cases [95%CI 81.8, 95.4] (Figure 2). Leave-one-out sensitivity analyses did not significantly change the pooled estimates obtained from the random-effects meta-analysis. The funnel plot of the effect sizes indicated possible publication bias (Supplement Figure S1). However, a Peter’s test did not reveal significant publication bias (*p* = 0.295). Imputing four effect sizes using the trim-and-fill method and re-analyzing the data using the imputed effect sizes decreased the pooled rate of successful control of bleeding to 80.5% [95%CI 73.5, 85.8]. Minimal between-study heterogeneity was noted in the meta-analysis (τ^2^ = 0.779; I^2^ = 0%, *p* = 0.891). 

### 3.3. Rate of Rebleeding

**Figure 3:**
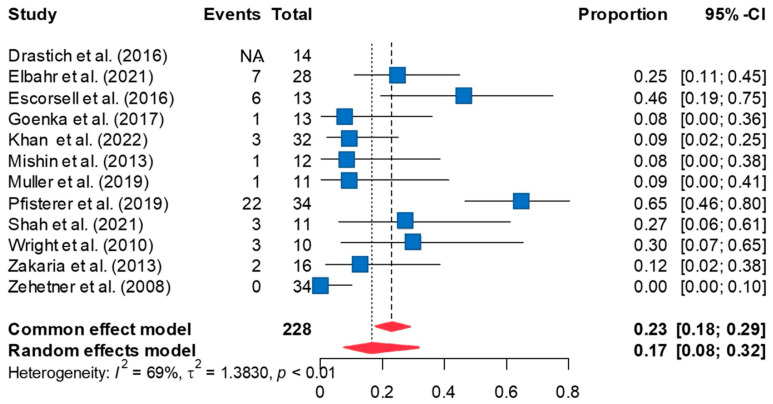
The forest plot of rate of rebleeding after SEMS placement demonstrates the rate of rebleeding is 16.6% [95%CI 7.9, 31.6] [8,10,11,12,20,21,22,23,24,25,26,27].

The overall rate of rebleeding in 11 studies was 16.6% based on the random-effects meta-analysis [95%CI 7.9, 31.6] (Figure 3). The duration between SEMS placement and rebleeding varied among the studies. Leave-one-out sensitivity analyses did not significantly affect the pooled rate of rebleeding observed in the meta-analysis. The funnel plot revealed possible publication bias (Supplement Figure S2). However, a Peter’s test did not reveal significant publication bias (*p* = 0.346). Imputing five effect sizes using the trim-and-fill method and re-analyzing the data using the imputed effect sizes increased the rate of rebleeding to 37.1% [95%CI 20.1, 58.0]. Significant between-study heterogeneity was high (τ^2^ = 1.383; I^2^ = 69%, *p* < 0.001). Univariate meta-regression analyses using year of publication, mean age, proportion of males, and duration of follow-up as possible covariates failed to explain this high between-study heterogeneity or to reveal any significant moderator effects.

### 3.4. Rate of Stent Ulceration

**Figure 4:**
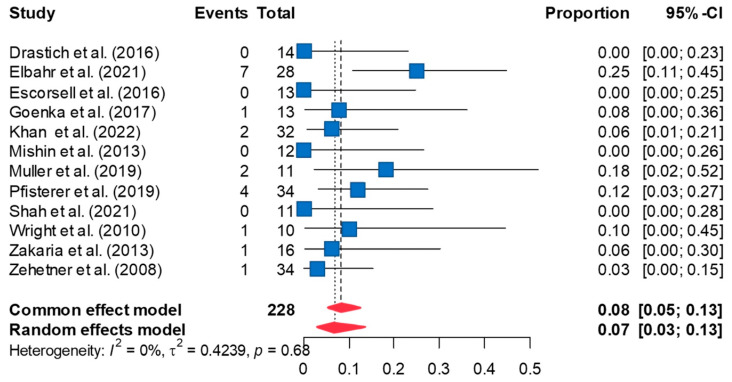
The forest plot of the rate of stent ulceration after SEMS placement demonstrates the rate of stent ulceration is 6.8% [95%CI 3.3, 13.3] [8,10,11,12,20,21,22,23,24,25,26,27].

The pooled rate of ulceration following esophageal SEMS placement was 6.8% [95%CI 3.3, 13.3] (Figure 4). The rate of ulceration remained consistent on leave-one-out sensitivity analyses. Peter’s test was not significant for publication bias (*p* = 0.980); however, the funnel plot was asymmetric, indicating possible publication bias (Supplement Figure S3). Trim-and-fill analysis imputed six additional effect sizes to restore funnel plot symmetry. Re-analyzing the data using these additional effect sizes increased the estimated rate of ulceration to 14.3% [95% CI 9.4, 21.2]. Between-study heterogeneity was low for this meta-analysis (τ^2^ = 0.424; I^2^ = 0%, *p* = 0.679).

### 3.5. Rate of Stent Migration

**Figure 5:**
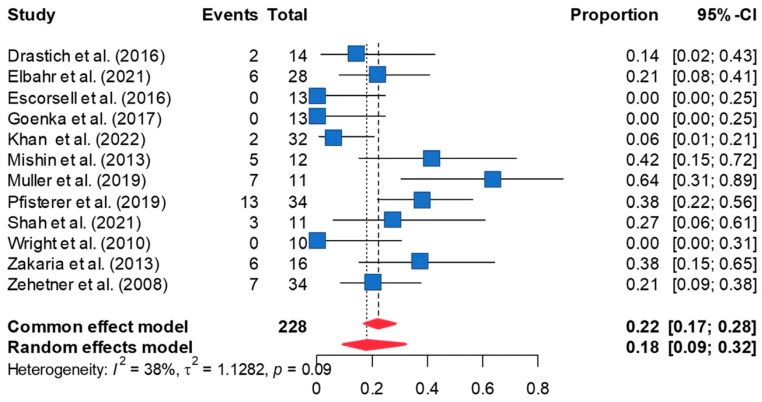
The forest plot showed the rate of stent migration in patients who underwent SEMS placement demonstrates the rate of stent migration is 18.2% [95%CI 9.4, 32.3] [8,10,11,12,20,21,22,23,24,25,26,27].

Inserted stents migrated in 18.2% of the cases in the random-effects meta-analysis [95%CI 9.4, 32.3] (Figure 5). This pooled estimated remained robust in leave-one-out sensitivity analyses. Peter’s test of publication bias was not significant (*p* = 0.243), and the funnel plot was relatively symmetric on visual inspection (Supplement Figure S4). However, trim-and-fill analysis imputed two additional effect sizes. Re-analyzing the data including these effect sizes increased the overall rate of stent migration to 28.4% [95%CI 18.9, 40.3]. Between-study heterogeneity revealed a statistical trend (τ^2^ = 1.128; I^2^ = 40%, *p* = 0.089). However, it did not explain meta-regression analyses, including year of publication, mean age, proportion of males, or duration of follow-up, and did not explain the residual heterogeneity or reveal a moderator effect.

### 3.6. Mortality Rate

**Figure 6:**
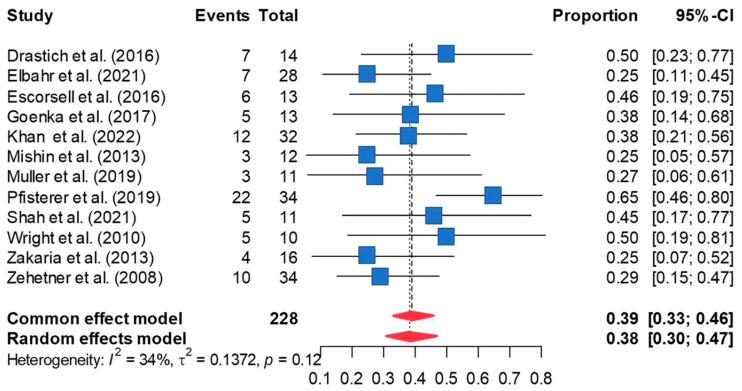
The forest plot of overall mortality in patients who underwent SEMS placement for acute refractory variceal bleeding demonstrates the overall mortality is 38.9% [95%CI 30.9, 47.6] [8,10,11,12,20,21,22,23,24,25,26,27].

A 38.9% mortality rate was observed in patients who underwent esophageal stenting [95%CI 30.9, 47.6] (Figure 6). The estimate was not significantly altered in leave-one-out sensitivity analyses. Publication bias was not a concern based on Peter’s test or funnel plot symmetry (Supplement Figure S5). Imputing one effect size using the trim-and-fill method and re-analyzing the data using the imputed effect size minimally increased the mortality estimate to 40.7% [95% CI 32.8, 49.1]. Between-study heterogeneity was not significant (τ^2^ = 0.138; I^2^ = 34%, *p* = 0.120).

## 4. Discussion

Our study demonstrates that esophageal SEMS placement is a feasible method for achieving hemostasis in patients with acute refractory variceal bleeding, defined as patients with active variceal bleeding that did not respond to pharmacologic and endoscopic therapy (Table 2). Self-expandable metal stents have a high successful rate in immediate control of bleeding up to 90.6% with infrequent adverse events following stent placement; the rate of rebleeding is 16.6%, the rate of stent ulceration is 6.8%, the rate of stent migration is 18.2%, and the overall mortality rate is 38.9%. 

Esophageal variceal bleeding is the third most common cause of upper-gastrointestinal bleeding following gastric ulcers and duodenal ulcers. Acute esophageal variceal bleeding is the most common cause of upper-gastrointestinal bleeding, which occurs in 60–70% in patients with cirrhosis and is a major complication of portal hypertension [1,28,29]. The lifetime incidence of esophageal varices in cirrhotic patients is as high as 80–90% [30]. In a patient who has a single episode of variceal bleeding, there is a 70% chance of rebleeding, and at least 30% of the rebleeding episodes are fatal. Failure to control variceal bleeding despite combined pharmacological and endoscopic therapy can occur in up to 10–20% of cases and is classified as refractory variceal bleeding. This condition is best managed by salvage polytetrafluoroethylene (PTFE)-covered TIPS, which directs portal blood flow directly into the hepatic vein, bypassing the liver, leading to a decrease in portal pressure [4,6,31]. However, TIPS might lead to hypoperfusion of liver parenchyma resulting in hepatic encephalopathy and deranged liver function. Therefore, the Baveno consensus developed a specific indication for placement of TIPS in acute refractory variceal bleeding in specific groups of patients; TIPS is indicated within 72 h if Child–Pugh class C < 14 points or Child–Pugh class B > 7 with active bleeding at initial endoscopy or hepatic venous pressure gradient (HVPG) > 20 mmHg at the time of hemorrhage [4]. In those with a Child–Pugh score ≥ 14 cirrhosis, or with a MELD score > 30, TIPS may be futile unless liver transplantation is available in the near term. Thus, the decision to perform TIPS should be taken on a case-by-case basis [4]. However, in some centers, TIPS might not be available as the procedure requires an interventional radiologist with a well-equipped center with anesthesiology.

With the need for expertise in the placement of TIPS, the more convenient and effective tools (prompt management) in managing acute refractory variceal bleeding with balloon tamponade and SEMSs are recommended as bridge therapy to a definitive treatment [4]. The traditional management of controlling acute refractory variceal bleeding with balloon tamponade that was first described in 1950 was widely used given the low cost and successful outcome in controlling the bleeding [32]. The success rate of control esophageal variceal bleeding with balloon tamponade using a Sengstaken–Blakemore tube was high, up to 91.5%; however, the balloon tamponade came with adverse events and may cause lethal complications, including aspiration, pressure necrosis, and esophageal rupture, which can occur in 6–20% of patients, and these occur more frequently with inexperienced staff [6]. There are other multiple-balloon tamponade strategies such as the Minnesota tube that is modified from the Sengstaken–Blakemore tube; however, the adverse events are similar. Therefore, other non-surgical techniques with lower adverse events in managing refractory esophageal variceal bleeding have been studied (or introduced). 

A non-surgical technique with SEMSs is effective and convenient, and, most importantly, the placement of SEMSs can be performed without radiological assistance or even an endoscope, since the device comes with delivery apparatus that has a built-in gastric balloon that guides stent placement [33]. The stent is removable, covered, and can be repositioned using the stent extractor. Self-expandable metal stents work by compressing varices after expansion in the lower esophagus, providing a tamponade effect similar to a Sengstaken–Blakemore tube, but have a lower rate of complication compared with the Sengstaken–Blakemore tube [6]. The endoscope is re-inserted after stent placement to confirm its position and efficacy in achieving hemostasis.

Even though the Sengstaken–Blakemore tube is cheaper than SEMSs, SEMSs are considered cost-effective tools for managing acute refractory variceal bleeding given their several advantages when compared with the Sengstaken–Blakemore tube. These include the duration of placement (7 days vs. within 24 h), comfort, the possibility of endoscopy and oral feeding, and lower aspiration/esophageal perforation rates as the stent has a security pressure valve that minimizes the risk of esophageal perforation [5,6]. This benefit in allowing oral intake helps improve liver function and the nutritional status of the patients to allow more durable therapy, i.e., TIPS or orthotopic liver transplantation. The stent can be left in place for up to two weeks and can be easily removed by endoscopy. Most of the studies included in our meta-analysis (11 out of 12) used the SEMS SX-Ella Danis stent (Ella-CS, Hradec Kralove, Czech Republic), which is 135 mm in length, has a 25 mm mid-diameter, and has a 30 mm end-diameter designed to tamponade bleeding varices in the distal esophagus [11,23]. The stent can be left in situ for up to 7 days.

A meta-analysis comparing the efficacy of SEMSs and TIPS in the treatment of refractory esophageal variceal bleeding included 21 studies (12 studies and 176 patients with SEMS placement vs. 9 studies and 398 patients with TIPS placement) reported a higher mortality rate in SEMSs with 43.6% (95%CI 28.6–59.8) and TIPS with 27.9 (95% CI 16.3–36); however, the mortality rate in TIPS group has high heterogeneity with a prediction interval (PI) of 2 to 88 and an I^2^ of 91 [34]. This study had the limitation in that the study only evaluated the mortality rate within the same group; SEMSs have a higher mortality rate and rebleeding rate when compared with TIPS [34]. Despite the rebleeding rate being higher in the SEMS group (19% vs. 9% in the TIPS group), the technical success rate in both groups is not much different (SEMS was 88%, and TIPS was 91%), but the availability of TIPS in emergencies becomes an issue due to limited resources and experienced radiologists. This study presents the results of SEMSs and TIPS side by side; however, our analysis has the limitation of retrospective comparison, and, therefore, we do not comment on the superiority and/or inferiority of one modality to the other.

Our study demonstrates that SEMSs can be a good tool for controlling bleeding immediately and can serve as a bridge to definitive therapy in acute refractory esophageal variceal bleeding. However, the following considerations seem important. First, in case of severe bleeding, TIPS should be placed in the readily available center with the exception of the patients with relative contraindications for TIPS, such as hepatocellular carcinoma and portal vein thrombosis. Second, the use of SEMSs is not readily available in all centers and is not familiar to every endoscopist, which may result in operator dependence and increase adverse events if the stent is placed by an inexperienced endoscopist. Even though stents come with a delivery apparatus that can easily be deployed without endoscopic guidance, in most studies, the endoscopist is the clinician who places the stent since stent dislocation or displacement and uncontrolled bleeding can occur and should be managed by the endoscopist. Other limitations of SEMSs include ulceration after the removal of the stent, stent migration causing irritation and uncomfortable sensation, and the need for follow-up imaging to confirm stent position and rate of rebleeding. The articles used in this study did not report the experience of the endoscopist, such as the number of years of practice, or the resources available at their facilities. The experience of the endoscopist might affect the position of stent placement and consequently bleeding control. Other limitations include the degree of cirrhosis, which varied in the studies used in this analysis, and in retrospective studies, critical information might not be available. We could not analyze patient-level data in terms of outcomes, differences in bleeding profile, cirrhotic stage, and underlying comorbidities which might lead to significant differences in outcome and differences in severity of variceal bleeding. In addition, the duration of stent placement time is different in the various studies, and this might influence the outcomes. The studies included in our study reported stent placement times that varied from 2 days to 17.5 days.

## 5. Conclusions

This updated meta-analysis supports the current guideline for managing acute refractory variceal bleeding and demonstrates that SEMSs can be used as a bridge therapy in acute refractory variceal bleeding in centers in which definitive therapy, such as TIPS and liver transplantation, is not readily available. Definitive therapy remains the most effective treatment and should be initiated promptly in centers with the required resources and expertise. The lack of studies on TIPS and liver transplantation following SEMS placement in acute refractory variceal bleeding limits our understanding of the overall benefit of SEMS placement.

## Figures and Tables

**Table 1:** Characteristics of studies evaluating the efficacy of self-expandable metal stents in acute refractory variceal bleeding.

Author	Year	Country	Publication Type	Study Type	Number of Patients	Mean Age	Male	Child–Pugh Score	MELD Score	Etiology of Cirrhosis
Drastich [20]	2016	Czech	Abstract	Retrospective	14	52.9	7	NR	NR	NR
Elbahr [12]	2021	Egypt	Full paper	Retrospective	28	57.8	24	A (3), B (15), C (10)	15.7 ± 6.3	HBV (24), HCV (4)
Escorsell [8]	2016	Spain	Full paper	Prospective	13	69.0 *	13	A (3), B and C (10)	16.5 (9–32) *	Alcohol (8), HCV (3), others (2)
Goenka [21]	2017	India	Full paper	Retrospective	12	53.0	11	NR	20.2 ± 6.0 (14–35)	Alcohol (4), HBV (1), HCV (3), NASH (1), others (3)
Khan [11]	2022	Australia	Full paper	Retrospective	30	53.3 *	20	NR	20.3 (7–40)	Alcohol (15), HBV (4), alcohol and HCV (3), HCV (1), NASH (3), others (4)
Mishin [22]	2013	Moldova	Abstract	Retrospective	12	46.9	8	NR	NR	NR
Muller [23]	2015	Germany	Full paper	Retrospective	11	64.2	8	A (1), B (6), C (3)	16.8 (8–36)	Alcohol (9), HBV (1), others (2)
Pfisterer [24]	2019	Austria	Full paper	Retrospective	34	55.5	28	A (1), B (10), C (8)	18 * IQR 10	Alcohol (16), viral hepatitis (8), alcohol and viral (4), others (6)
Shah [10]	2021	USA	Abstract	Retrospective	11	58.8	8	NR	NR	Alcohol (5), HCV (1), NASH (4), other (1)
Wright [25]	2010	UK	Full paper	Case series	10	49.4	9	NR	32 (23–39) *	Alcohol (6), alcohol and HCV (2), others (2)
Zakaria [26]	2013	Egypt	Full paper	Prospective	16	55.6	14	A (2), B (8), C (6)	NR	NR
Zehetner [27]	2008	Austria	Full paper	Retrospective	34	56.0	33	A (0), B (13), C (21)	NR	Alcohol (26), viral hepatitis (4), others (4)

* Median. NR, not reported. HBV, hepatitis B virus. HCV, hepatitis C virus. NASH, nonalcoholic steatohepatitis.

**Table 2:** The efficacy and adverse events of self-expandable metal stents in acute refractory variceal bleeding.

Author	Year	SEMS Type	Number of Patients	Successful Control of Bleeding	Stent Migration	Ulceration	Rebleeding	Mortality	Success of Deployment	Follow-Up(Days)	Stent Indwell Time (Days)
Drastich [20]	2016	SX-ELLA Danis	14	11	2	0	NR	7	9	180	9.5
Elbahr [12]	2021	NITI-S Mega stents-Tae Wong-S Korea	28	23	6	7	7	7	NR?	42	NR
Escorsell [8]	2016	SX-ELLA Danis	13	11	0	0	6	6	12	42	7
Goenka [21]	2017	SX-ELLA Danis	12 *	13	0	1	1	5	13	30	17.5
Khan [11]	2022	SX-ELLA Danis	30	31	2	2	3	12	31 (from 32 stents)	42	6.4
Mishin [22]	2013	SX-ELLA Danis	12	12	5	0	1	3	12	30	NR
Muller [23]	2015	SX-ELLA Danis	11	11	7	2	1	3	11	42	12.1
Pfisterer [24]	2019	SX-ELLA Danis	34	27	13	4	22	22	NR	42	5
Shah [10]	2021	NR	11	8	3	0	3	5	NR	42	13.4
Wright [25]	2010	SX-ELLA Danis	10	7	0	1	3	5	9	42	9
Zakaria [26]	2013	SX-ELLA Danis	16	14	6	1	2	4		NR	2–4
Zehetner [27]	2008	SX-ELLA Danis	34	34	7	1	0	10	34	60	5

NR, not reported. * 12 patients but 13 stents placed (13 procedures).

## Data Availability

Not applicable.

## Supplementary Materials

The following supporting information can be downloaded at: https://www.mdpi.com/article/10.3390/jcm13020357/s1, Figure S1: Funnel plot of SEMs in immediate bleeding control; Figure S2: Funnel plot of SEMs and rate of rebleeding; Figure S3: Funnel plot of SEMs and rate of stent ulceration; Figure S4: Funnel plot of SEMs and rates of stent migration; Figure S5: Funnel plot of SEMs and overall mortality rate; Table S1: Table demonstrates a meta-regression; Table S2: The Newcastle-Ottawa quality assessment scale of the included cohort studies.
