# Supplementary material for: Esophageal Stent in Acute Refractory Variceal Bleeding: A Systematic Review and a Meta-Analysis
Source: J Clin Med. 2024 Jan 9;13(2):357. doi: 10.3390/jcm13020357 (PMC10816372; doi:10.3390/jcm13020357)
Supplement: Supplementary file 1 [file jcm-13-00357-s001.zip › jcm-2801969-supplementary.pdf]

Supplementary Materials:

Supplementary Figures

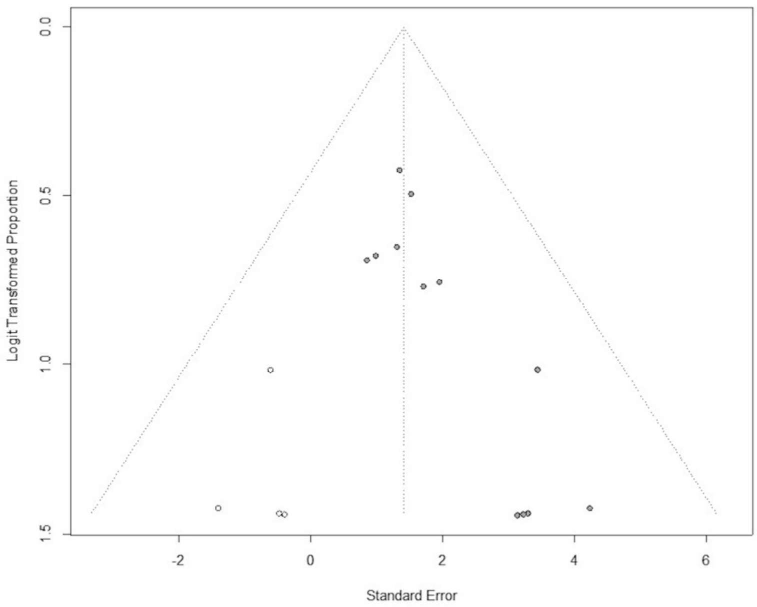

Supplementary Figure S1: Funnel plot of SEMS in immediate bleeding control.

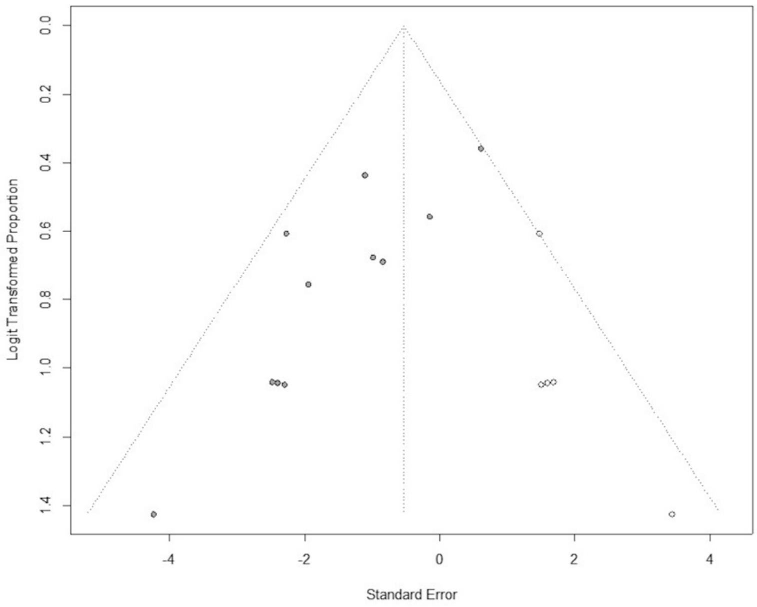

Supplementary Figure S2: Funnel plot of SEMS and rate of rebleeding.

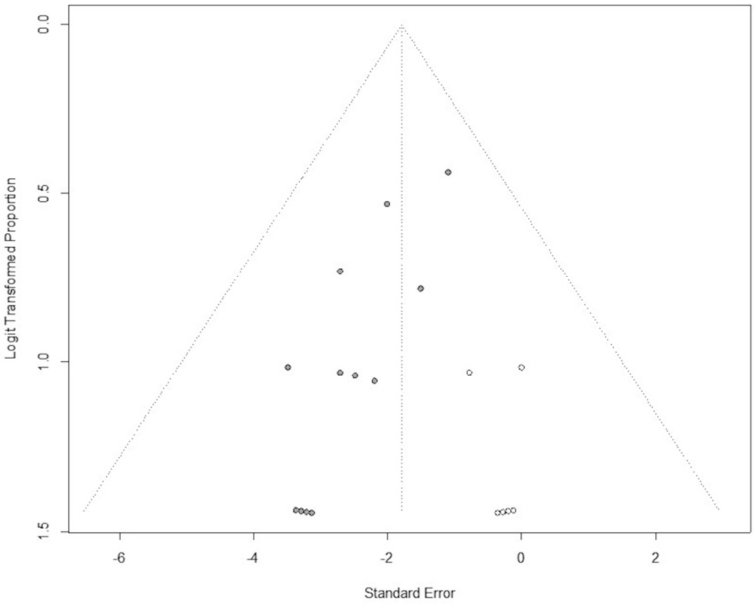

Supplementary Figure S3: Funnel plot of SEMS and rate of stent ulceration.

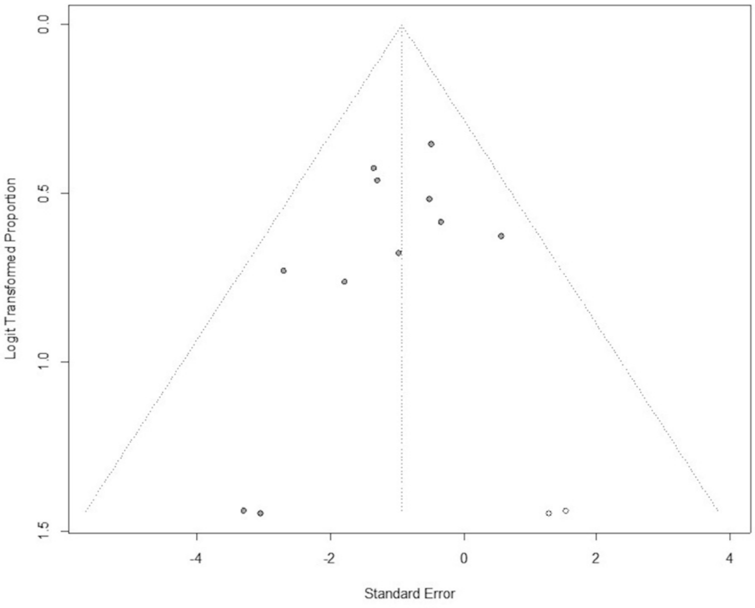

Supplementary Figure S4: Funnel plot of SEMS and rates of stent migration.

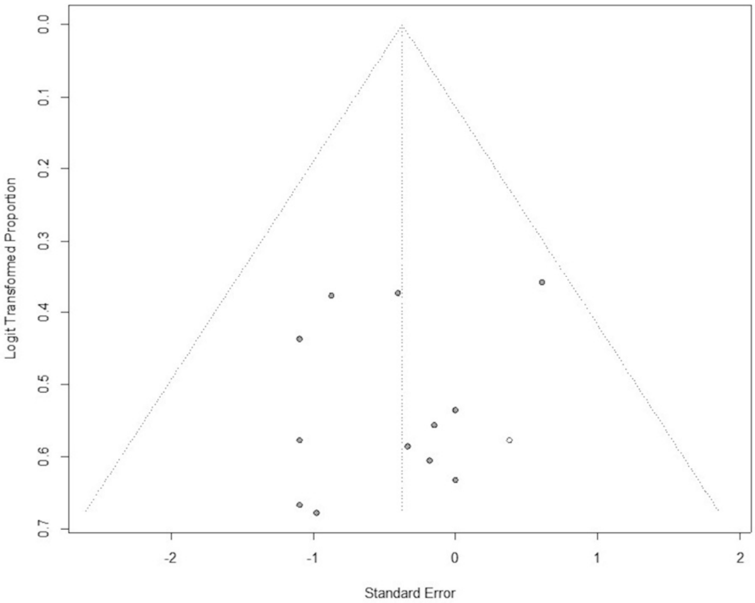

Supplementary Figure S5: Funnel plot of SEMS and overall mortality rate.

Supplementary Table

Supplementary Table S1: Table demonstrates a meta-regression.

Dependent Variable	Model	Independent Variable	β	SE	z-statistic	p-value
Rebleeding	1	Intercept	-244.829	184.9891	-1.3235	0.1857
		Year	0.1206	0.0917	1.315	0.1885
		k = 11, τ2 = 1.178, I2 = 74%, p < 0.001				
	2	Intercept	-5.2042	3.904	-1.3331	0.1825
		Age	0.0638	0.0685	0.9306	0.3521
		k = 11, τ2 = 1.124, I2 = 75%, p < 0.001				
	3	Intercept	-1.5207	0.9272	-1.64	0.101
		Male	-0.0057	0.0506	-0.1118	0.911
		k = 11, τ2 = 1.394, I2 = 76%, p < 0.001				
	4	Intercept	0.5052	2.6981	0.1873	0.8515
		Follow-up	-0.0504	0.0649	-0.7763	0.4376
		k = 10, τ2 = 1.477, I2 = 80%, p < 0.001				
Stent migration	5	Intercept	-42.0163	169.5924	-0.2477	0.8043
		Year	0.0201	0.0841	0.2389	0.8112
		k = 12, τ2 = 1.136, I2 = 74%, p < 0.001				
	6	Intercept	-2.3817	3.8294	-0.622	0.534
		Age	0.0158	0.0681	0.2319	0.8166
		k = 12, τ2 = 1.109, I2 = 76%, p < 0.001				
	7	Intercept	-1.6344	0.7934	-2.0599	0.0394
		Male	0.0081	0.0414	0.1954	0.845
		k = 12, τ2 = 1.138, I2 = 75%, p < 0.001				
	8	Intercept	-1.522	0.7158	-2.1263	0.0335
		Follow-up	-0.0019	0.0103	-0.1815	0.856
		k = 11, τ2 = 1.266, I2 = 77%, p < 0.001				

Supplementary Table S2: The Newcastle-Ottawa quality assessment scale of the included cohort studies.

	Selection				Comparability	Outcome			Total score
	Representativeness	Selection of the non-exposed cohort	Ascertainment	Endpoint not presented at start	Comparability (confounding)	Assessment of outcome	Follow-up duration	Adequacy of follow-up	
Drastich	★	★	★	★	★	★	★	0	7
Elbahr	★	★	★	★	★	★	★	★	8
Escorsell	★	★	★	★	★	★	★	★	8
Goenka	★	★	★	★	★	★	★	★	8
Khan	★	★	★	★	★	★	★	★	8
Mishin	★	★	★	★	★	0	★	★	7
Muller	★	★	★	★	★	★	★	★	8
Pfisterer	★	★	★	★	★	★	★	★	8
Shah	★	★	★	★	★	★	★	★	8
Wright	★	★	★	★	★	★	★	★	8
Zakaria	★	★	★	★	★	★	0	★	7
Zehetner	★	★	★	★	★	★	★	★	8
